# Clinical characteristics and antimicrobial susceptibilities of viridans streptococcal bacteremia during febrile neutropenia in patients with hematologic malignancies: a comparison between adults and children

**DOI:** 10.1186/1471-2334-13-273

**Published:** 2013-06-17

**Authors:** Seung Beom Han, E Young Bae, Jae Wook Lee, Dong-Gun Lee, Nack-Gyun Chung, Dae-Chul Jeong, Bin Cho, Jin Han Kang, Hack-Ki Kim

**Affiliations:** 1 Department of Pediatrics, College of Medicine, The Catholic University of Korea, Seoul, Republic of Korea; 2 Vaccine Bio Research Institute, College of Medicine, The Catholic University of Korea, Seoul, Republic of Korea; 3 The Catholic Blood and Marrow Transplantation Center, College of Medicine, The Catholic University of Korea, Seoul, Republic of Korea; 4 Division of Infectious Diseases, Department of Internal Medicine, College of Medicine, The Catholic University of Korea, Seoul, Republic of Korea

**Keywords:** Viridans streptococci, Bacteremia, Neutropenia, Fever

## Abstract

**Background:**

This study was performed to compare the clinical characteristics and antibiotic susceptibilities of viridans streptococcal bacteremia (VSB) between febrile neutropenic adults and children with hematologic malignancies.

**Methods:**

The consecutive medical records of neutropenic patients with hematologic malignancies who were admitted to the Catholic Blood and Marrow Transplantation Center between April 2009 and July 2012, and who were subsequently diagnosed with VSB were reviewed retrospectively. A comparison was made between the clinical and laboratory characteristics of adults and pediatric patients and also between patients with cefepime susceptible or not susceptible VSB.

**Results:**

A total of 202 episodes (141 in adults, 61 in children) of VSB were identified. Among them, 26 (12.9%) cases had severe complications including four (2.0%) cases of death attributable to VSB. For antibacterial prophylaxis, most adults received ciprofloxacin (97.1%), but children more frequently received trimethoprim/sulfamethoxazole (86.9%). Oral mucositis (*p* = 0.005) and abdominal pain (*p* = 0.001) were found more frequently in adults, and cough was found more frequently in children (*p* = 0.004). The occurrence rates of severe complications and death attributable to VSB were not significantly different between adults and children. Susceptibility rate to cefepime was significantly higher in adults than children (85.7% vs. 66.1%, *p* = 0.002). However, in multivariate analysis, cefepime susceptibility had no impact on clinical outcome.

**Conclusions:**

There was no significant difference in clinical outcome between adults and children with VSB despite a difference in cefepime susceptibility. Hence, different antibiotic treatment strategies may not be necessary.

## Background

Bacteremia is identified in 10-27% of febrile neutropenic patients with hematologic malignancies [1-3], and 18-29% of the bacteremia is caused by viridans streptococci [1,4,5]. Although Gram negative bacteria were the most common isolates to cause bacteremia in febrile neutropenic patients in the past [6], viridans streptococci are currently one of the most common isolates in both adults and children [1,4,5,7].

Viridans streptococcal bacteremia (VSB) has been reported to cause severe complications such as shock and acute respiratory distress syndrome (ARDS) in 18-39% of infected neutropenic patients and death in up to 20% [8-10]. A higher occurrence rate of these severe complications was reported in children compared to adults [11].

Although the Infectious Diseases Society of America (IDSA) and Korean guidelines state that β-lactam antibiotics are adequate for viridans streptococcal infections [2,12], it is uncertain whether the same practice guidelines can be applied to treat infections in adults and children because of the different complication frequencies [11] and the potentially different antibiotic susceptibilities to viridans streptococci in febrile neutropenic adults and children with VSB.

We performed this retrospective study to compare clinical characteristics including the occurrence of severe complications and antibiotic susceptibilities of viridans streptococci between febrile neutropenic adults and children with hematologic malignancies, and to propose appropriate antibacterial treatment strategies for adults and children.

## Methods

### Study design

The consecutive medical records of patients diagnosed with VSB during febrile neutropenia were reviewed retrospectively. The patients were admitted to the Catholic Blood and Marrow Transplantation (BMT) Center between April 2009 and July 2012, and received conventional chemotherapy or hematopoietic cell transplantation (HCT) for their hematologic malignancies. The Catholic BMT Center is affiliated with Seoul St. Mary’s Hospital in Seoul, Republic of Korea and is a university-affiliated tertiary center with about 1,300 beds. There are separate hematology wards for adults and children, and the adult hematology ward consists of separate wards for intensive conventional chemotherapy and HCT patients. The Institutional Review Board (IRB) of Seoul St. Mary’s Hospital approved this research protocol with a waiver of informed consent (KC12RISI0607, approved on September 24, 2012).

### Patients and data collection

Patients who were younger than 20 years were categorized as children, and the rest as adults according to the IRB guideline, and clinical and laboratory characteristics and antibiotic susceptibilities were compared between the adults and children. The same clinical and laboratory characteristics were also compared between patients with VSB susceptible to cefepime, one of the empirical antibiotics used for febrile neutropenic patients, and those with VSB not susceptible to cefepime. Data gathered on patients’ demographics and clinical characteristics consisted of gender, underlying disease with remission status, type of therapy preceding febrile neutropenia, number of days from the beginning of respective therapies to the diagnosis of VSB, use of antibacterial prophylaxis, and occurrence of oral mucositis, respiratory symptoms, gastrointestinal symptoms, severe complications and polymicrobial infection by other bacteria or fungi. Laboratory characteristics consisted of white blood cell (WBC) count and absolute neutrophil count (ANC) upon the diagnosis of VSB, the number of neutropenic days before the diagnosis of VSB, total number of neutropenic days during the febrile neutropenic episode, and the peak C-reactive protein (CRP) level within a week after the diagnosis of VSB.

Ceftazidime or cefepime with aminoglycoside, and piperacillin/tazobactam with aminoglycoside were administered as initial empirical antibacterial therapy for febrile neutropenia in adults and children, respectively. After three to five days of initial antibacterial therapy, an adjustment, if needed, was made according to the Korean guideline for febrile neutropenia [12]. Glycopeptides were given based on the indications recommended by the Korean guideline [12].

### Antibiotic susceptibility test

Blood for culture was sampled using sterile technique with one set from a peripheral vein and another set from a central catheter. In adults, 10–15 mL of blood was inoculated into each aerobic and anaerobic culture bottle (BD BACTEC™ Plus Aerobic/F, Lytic/10 Anaerobic/F Culture Vials, Becton Dickinson, Sparks, MD, USA), and in children, 1–3 mL of blood was inoculated into a culture bottle (BD BACTEC™ Peds Plus Culture Vial, Becton Dickinson, Sparks, MD, USA). The bottles were immediately transported to the clinical microbiology laboratory. Automated culture systems were used to detect bacterial growth (BACTEC™ FX, Becton Dickinson, Sparks, MD, USA) and to identify the exact bacterial type (VITEK®2, BioMériux, Hazelwood, MO, USA). Antibiotic susceptibility tests were performed on a Muller-Hinton agar plate with 5% sheep blood, according to the Clinical and Laboratory Standards Institute (CLSI) recommendations [13]. The susceptibilities were determined by using an E-test for penicillin and cefotaxime, and using a disk diffusion method for cefepime, erythromycin, clindamycin, vancomycin, and linezolid. A result of ‘S’ was considered susceptible, and results of ‘I’ and ‘R’ were considered not susceptible. Susceptibility rates to each antibiotic drug were calculated and compared between adults and children. Because antibiotic susceptibilities and clinical characteristics were not significantly different among viridans streptococcal species [5,9], we did not identify the different species of viridans streptococci.

### Definitions

VSB was defined as growth of viridans streptococci from at least one peripheral or central blood sample. Neutropenia was defined as having an ANC lower than 500/μL or an ANC lower than 1,000/μL that was predicted to be lower than 500/μL within two to three days, and fever was defined as a body temperature higher than 38.0°C with a tympanic thermometer or 37.5°C with an axillary thermometer [12]. Severe complications included shock, any kind of mechanical ventilator care, ARDS, and death. Shock was defined as hypotension (mean arterial pressure less than 60 mmHg in adults, and systolic blood pressure less than the 5^th^ percentile to age in children) requiring an intravenous fluid bolus or inotropic agents to maintain normal blood pressure [8,14,15], and ARDS was defined as PaO_2_/FiO_2_ < 200 in arterial blood gas analysis of a patient with hypoxia of SpO_2_ < 90% and bilateral pulmonary infiltrates on the chest X-ray [16]. The severe complications were considered to be attributable to VSB if there was no clinical improvement after the diagnosis of VSB with severe complications, no other infectious isolates were detected, no deterioration in underlying malignancy was observed, and no other clinical diagnoses were made. Death attributable to VSB was defined as death accompanied by severe complications attributable to VSB within 14 days after the diagnosis of VSB, and overall death included death from all causes within a month after the diagnosis of VSB [17].

### Statistical analysis

Statistical analysis was performed with SPSS Statistics 17.0 (SPSS Inc., Chicago, IL, USA), and statistical significance was defined as a two-sided *p* < 0.05. In comparisons between adults and children and patients with VSB susceptible and not susceptible to cefepime, a Student’s t-test was used for numerical variables, and a χ^2^ test was used for categorical variables. Multivariate analysis using multiple logistic regression tests was performed for statistically significant factors derived from univariate analysis to determine factors related to the susceptibility of viridans streptococci to cefepime. The peak CRP level within a week after the diagnosis of VSB, predicting the development of severe complications attributable to VSB, was determined by a receiver operating characteristic (ROC) curve.

## Results

### Epidemiology of viridans streptococcal bacteremia in febrile neutropenic adults and children with hematologic malignancies

During the study period, there were 2,677 admissions in 1,248 adults and 4,219 admissions in 511 children for conventional chemotherapy, HCT, or febrile neutropenia following chemotherapy. In adults, 745 episodes of bacteremia in 487 patients and 141 episodes of VSB in 134 patients were identified, and the incidence of bacteremia and VSB were 9.17 and 1.74 episodes per 1,000 person-days, respectively. In children, 301 episodes of bacteremia in 162 patients and 61 episodes of VSB in 54 patients were identified, and the incidence of bacteremia and VSB were 6.64 and 1.35 episodes per 1,000 person-days, respectively. Among the total 202 episodes of VSB in adults and children, 42 (20.8%) cases with severe complications including 14 (6.9%) deaths were identified, and 26 (12.9%) of them, including four (2.0%) deaths were attributable to VSB. The other cases leading to mortality were due to uncontrolled underlying hematologic malignancies. Multiple episodes of VSB were diagnosed in 11 patients. Eight patients (five adults and three children) each experienced two episodes, and three patients (one adult and two children) each experienced three episodes of VSB. Each episode was diagnosed during separate admissions. None of the patients experienced multiple episodes of severe complications.

### Comparison of clinical and laboratory characteristics between adults and children

Among the total 202 cases of VSB, 108 (53.5%) cases were male, and 147 (72.8%) cases suffered from acute myeloid leukemia (AML). VSB occurred a median of 12 days (inter quartile range, IQR: 10–14) after the preceding therapy. Conventional chemotherapy and HCT accounted for 95.0% and 5.0% of the preceding therapy, respectively. Diarrhea (60/202, 29.7%) was the most common symptom accompanying fever, and was followed by oral mucositis (41/202, 20.3%) and abdominal pain (38/202, 18.8%).

More children were male compared to the adult group (*p* = 0.010, Table 1). AML accounted for about 70% of the underlying diseases in both adults and children, and the distribution of underlying diseases was not significantly different between the two groups (Table 1). All children with VSB had been treated with conventional chemotherapy, whilst 92.9% of adults had been treated with conventional chemotherapy, and 7.1% with HCT. The type of preceding therapy was not significantly different between the two groups (Table 1). The median number of days from the beginning of the preceding therapy to the diagnosis of VSB was 12 days (IQR: 10–13) in adults and 13 days (IQR: 12–14) in children, and this difference was statistically significant (*p* < 0.001, Table 1). Antibacterial prophylaxis was administered to 194 (96.0%) patients; 141 (69.8%) patients received ciprofloxacin (500 mg twice a day), and 53 (26.2%) patients received trimethoprim/sulfamethoxazole (TMP/SMX, trimethoprim 150 mg/m^2^ once a day, three times a week). Five (2.5%) patients did not receive any antibacterial prophylaxis, and the other three (1.5%) patients experienced VSB during antibiotic treatment with ceftazidime given due to preceding febrile neutropenia. Adults received ciprofloxacin more frequently (97.1%), and children received TMP/SMX more frequently (86.9%, Table 1). This difference in antibacterial prophylaxis occurred because fluoroquinolones are not recommended to children aged less than 18 years in Korea due to the risk of skeletal abnormalities. The seven patients in the pediatric group who received ciprofloxacin prophylaxis were older than 18 years. Among the symptoms accompanying VSB, oral mucositis (*p* = 0.005) and abdominal pain (*p* = 0.001) were more common in adults, and cough was more common in children (*p* = 0.004, Table 1). The occurrence rates of severe complications attributable to VSB, overall mortality, and mortality attributable to VSB were not significantly different between adults and children (Table 1). CRP levels were measured a median of three times (IQR: 3–3) within the first week after the diagnosis of VSB, and the peak CRP levels within a week were detected a median of four days (IQR: 3–5) after the diagnosis of VSB. The frequency of measuring CRP levels and the time of the peak CRP levels were not significantly different between adults and children. There was no significant difference in laboratory results between adults and children (Table 1).

**Table 1:** Comparison between adults and children with viridans streptococcal bacteremia during febrile neutropenia

**Factor**	**Adults (≥ 20 years old) (n=141)**	**Children (< 20 years old) (n=61)**	***p*-value**
Age (years)	44 (36-54)	9 (3-15)	
Gender (male)	67 (47.5)	41 (67.2)	**0.010**
Underlying disease			0.215
Acute myeloid leukemia	103 (73.1)	44 (72.1)	
Acute lymphoblastic leukemia	23 (16.3)	13 (21.3)	
Other leukemias	4 (2.8)	3 (4.9)	
Non-Hodgkin lymphoma	8 (5.7)	1 (1.6)	
Multiple myeloma	3 (2.1)	0 (0.0)	
Complete remission status	84 (59.6)	40 (65.6)	0.421
Preceding therapy			0.103
Conventional chemotherapy	131 (92.9)	61 (100.0)	
Hematopoietic cell transplantation			
Autologous	8 (5.7)	0 (0.0)	
Allogeneic	2 (1.4)	0 (0.0)	
Duration after the preceding therapy (days)	12 (10-13)	13 (12-14)	**<0.001**
Antibacterial prophylaxis^*^			**< 0.001**
None	4 (2.9)	1 (1.6)	
Trimethoprim/sulfamethoxazole	0 (0)	53 (86.9)	
Ciprofloxacin	134 (97.1)	7 (11.5)	
Accompanying symptoms			
Oral mucositis	36 (25.5)	5 (8.2)	**0.005**
Cough	9 (6.4)	12 (19.7)	**0.004**
Sputum	5 (3.5)	4 (6.6)	0.458
Rhinorrhea	1 (0.7)	0 (0.0)	1.000
Diarrhea	46 (32.6)	14 (23.0)	0.167
Abdominal pain	35 (24.8)	3 (4.9)	**0.001**
Polymicrobial infection			
Other bacterial infection	18 (12.8)	4 (6.6)	0.193
Fungal infection	16 (11.3)	2 (3.3)	0.065
Complications attributable to VSB	22 (15.6)	4 (6.6)	0.078
Ventilator care	5 (3.5)	3 (4.9)	0.700
Acute respiratory distress syndrome	4 (2.8)	2 (3.3)	1.000
Shock	22 (15.6)	4 (76.6)	0.078
Death	3 (2.1)	1 (1.6)	1.000
Overall death	12 (8.5)	2 (3.3)	0.236
Laboratory findings			
White blood cell count (/μL)	50 (20-160)	60 (30-135)	0.127
Absolute neutrophil count (/μL)	0 (0-0)	0 (0-0)	0.228
Duration of neutropenia before the diagnosis of VSB (days)	6 (4-7)	6 (4-8)	0.138
Total duration of neutropenia (days)	17 (12-24)	15 (11-22)	0.823
Peak C-reactive protein level (mg/dL)	19.6 (13.2-26.5)	18.6 (9.7-25.5)	0.157

Data are median (inter quartile range) or No. (%) of cases.

*VSB* viridans streptococcal bacteremia.

^*^Antibacterial prophylaxis was evaluated in 199 patients (138 adults, 61 children) except 3 adults who had been undergoing ceftazidime treatment.

### Comparison of antibiotic susceptibilities between adults and children

Antibiotic susceptibility was assessed in 201/202 (99.5%) of bacterial isolates, that is, all the isolates except for one from an adult patient (Table 2). In adults, the susceptibility rate to each antibiotic was: penicillin 57/140 (40.7%), cefotaxime 127/140 (90.7%), cefepime 120/140 (85.7%), vancomycin 140/140 (100%), linezolid 140/140 (100%), clindamycin 121/140 (86.4%), and erythromycin 78/140 (55.7%). In children, the susceptibility rates were: penicillin 22/61 (36.1%), cefotaxime 39/60 (65.0%), cefepime 39/59 (66.1%), vancomycin 61/61 (100%), linezolid 60/61 (98.4%), clindamycin 51/61 (83.6%), and erythromycin 21/61 (34.4%). The susceptibility rates to cefotaxime, cefepime, and erythromycin were significantly higher in adults than in children (Table 2).

**Table 2:** Comparison of antibiotic susceptibility rates between adults and children

**Antibiotics**	All patients^*^(n=201)	**Adults (≥ 20 years old) (n=140)**	**Children (< 20 years old) (n=61)**	***p*-value**
Penicillin	79/201 (39.3)	57/140 (40.7)	22/61 (36.1)	0.535
Cefotaxime^†^	166/200 (83.0)	127/140 (90.7)	39/60 (65.0)	**< 0.001**
Cefepime^‡^	159/199 (79.9)	120/140 (85.7)	39/59 (66.1)	**0.002**
Vancomycin	201/201 (100.0)	140/140 (100.0)	61/61 (100.0)	NA
Linezolid	200/201 (99.5)	140/140 (100.0)	60/61 (98.4)	0.303
Clindamycin	172/201 (85.6)	121/140 (86.4)	51/61 (83.6)	0.601
Erythromycin	99/201 (49.3)	78/140 (55.7)	21/61 (34.4)	**0.006**

Data are No. (%) of cases.

*NA* not available.

^*^Antibiotic susceptibility tests were performed in 201 patients (140 adults, 61 children).

^†^Antibiotic susceptibility tests were performed in 200 patients (140 adults, 60 children).

^‡^Antibiotic susceptibility tests were performed in 199 patients (140 adults, 59 children).

### Comparison between patients with severe complications attributable to viridans streptococcal bacteremia and those without severe complications

The median of peak CRP levels within a week after the diagnosis of VSB was 27.2 mg/dL (IQR: 21.4-33.7) in patients with severe complications attributable to VSB and 17.8 mg/dL (IQR: 11.8-25.5) in those without severe complications. These were significantly different (*p* < 0.001). Peak CRP levels were detected a median of four days (IQR: 3–5) after the diagnosis of VSB in the two groups without a significant difference. The cut-off value of the peak CRP level predicting the development of severe complications attributable to VSB was determined using an ROC curve as 21.0 mg/dL (Area under the curve = 0.772) with sensitivity, specificity, positive predictive value, and negative predictive value of 77%, 62%, 23%, and 95%, respectively. There were no other significant differences in clinical and laboratory characteristics between the two groups. The antibiotic susceptibility rate of each antibiotic drug was not significantly different between the two patient groups (Table 3).

**Table 3:** Antibiotic susceptibility rates according to the occurrence of severe complications attributable to viridans streptococcal bacteremia

**Antibiotics**	All patients^*^(n=201)	**Without complications (n=175)**	**With complications (n=26)**	***p*-value**
Penicillin	79/201 (39.3)	70/175 (40.0)	9/26 (34.6)	0.600
Cefotaxime^†^	166/200 (83.0)	142/174 (81.6)	24/26 (92.3)	0.263
Cefepime^‡^	159/199 (79.9)	136/173 (78.6)	23/26 (88.5)	0.243
Vancomycin	201/201 (100.0)	175/175 (100.0)	26/26 (100.0)	NA
Linezolid	200/201 (99.5)	174/175 (99.4)	26/26 (100.0)	1.000
Clindamycin	172/201 (85.6)	151/175 (86.3)	21/26 (80.8)	0.548
Erythromycin	99/201 (49.3)	86/175 (49.1)	13/26 (50.0)	0.935

Data are No. (%) of cases.

*NA* not available.

^*^Antibiotic susceptibility tests were performed in 201 patients (175 without severe complications, 26 with severe complications).

^†^Antibiotic susceptibility tests were performed in 200 patients (174 without severe complications, 26 with severe complications).

^‡^Antibiotic susceptibility tests were performed in 199 patients (173 without severe complications, 26 with severe complications).

### Comparison between patients with viridans streptococcal bacteremia susceptible to cefepime and not susceptible to cefepime

Susceptibility tests to cefepime were conducted in 199 isolates, and 159 (79.9%) isolates were susceptible to cefepime (Table 4). In univariate analysis, patients with VSB susceptible to cefepime were older (*p* = 0.005), more likely to be in complete remission status (*p* = 0.037), more likely to have received ciprofloxacin prophylaxis (*p* < 0.001), and had a longer duration of neutropenia before the diagnosis of VSB (*p* = 0.021) than patients with VSB not susceptible to cefepime (Table 4). However, there was no significant factor related to cefepime susceptibility in multivariate analysis (Table 5).

**Table 4:** Comparison between patients with viridans streptococcal bacteremia susceptible to cefepime and those not susceptible to cefepime

**Factor**	**Susceptible to cefepime (n=159)**	**Not susceptible to cefepime (n=40)**	***p*-value**
Age (years)	38 (20-51)	17 (2-51)	**0.013**
Gender (male)	89 (56.0)	19 (47.5)	0.456
Underlying disease			0.320
Acute myeloid leukemia	117 (73.6)	28 (70.0)	
Acute lymphoblastic leukemia	26 (16.4)	9 (22.5)	
Other leukemias	6 (3.1)	1 (2.5)	
Non-Hodgkin lymphoma	8 (5.0)	1 (2.5)	
Multiple myeloma	2 (1.3)	1 (2.5)	
Complete remission status	104 (65.4)	19 (47.5)	**0.037**
Preceding therapy			0.732
Conventional chemotherapy	151 (95.0)	38 (95.0)	
Hematopoietic cell transplantation			
Autologous	6 (3.8)	2 (5.0)	
Allogeneic	2 (1.3)	0 (0.0)	
Duration after the preceding therapy (days)	12 (10-14)	13 (10-13)	0.568
Antibacterial prophylaxis^*^			**<0.001**
None	4 (2.5)	1 (2.7)	
Trimethoprim/sulfamethoxazole	31 (19.5)	20 (54.1)	
Ciprofloxacin	124 (78.0)	16 (43.2)	
Probability of antibacterial therapy after chemotherapy^†^ (%)	84.0 ± 29.6	81.8 ± 32.0	0.864
Previous antibacterial therapy for febrile neutropenia^†^ (times)	1 (1-2)	2 (1-2)	0.284
Accompanying symptoms			
Oral mucositis	35 (22.0)	6 (15.0)	0.327
Cough	18 (11.3)	2 (5.0)	0.377
Sputum	6 (3.8)	3 (7.5)	0.388
Rhinorrhea	1 (0.6)	0 (0.0)	1.000
Diarrhea	50 (31.4)	8 (20.0)	0.154
Abdominal pain	34 (21.4)	4 (10.0)	0.102
Polymicrobial infection			
Other bacterial infection	17 (10.7)	5 (12.5)	0.779
Fungal infection	16 (10.1)	2 (5.0)	0.537
Complications attributable to VSB	23 (14.5)	3 (7.5)	0.243
Ventilator care	8 (5.0)	0 (0.0)	0.362
Acute respiratory distress syndrome	6 (3.8)	0 (0.0)	0.602
Shock	23 (14.5)	3 (7.5)	0.243
Death	4 (2.5)	0 (0.0)	0.585
Overall death	13 (8.2)	1 (2.5)	0.309
Laboratory findings			
White blood cell count (/μL)	60 (20-160)	40 (20-118)	0.194
Absolute neutrophil count (/μL)	0 (0-0)	0 (0-0)	0.915
Duration of neutropenia before the diagnosis of VSB (days)	5 (4-7)	7 (5-11)	**0.021**
Total duration of neutropenia (days)	16 (12-23)	20 (12-24)	0.094
Peak C-reactive protein level (mg/dL)	19.6 (13.1-26.1)	17.1 (9.3-28.1)	0.397

Data are median (inter quartile range), mean ± SD, or No. (%) of cases.

*VSB* viridans streptococcal bacteremia.

^*^Antibacterial prophylaxis was evaluated in 196 patients (159 with viridans streptococcal bacteremia susceptible to cefepime, 37 with viridans streptococcal bacteremia not susceptible to cefepime) except 3 patients who had been undergoing ceftazidime treatment and 3 patients in whom cefepime susceptibility tests were not done.

^†^Information on previous chemotherapies and antibacterial therapies was completely reviewed in 166 patients (137 with viridans streptococcal bacteremia susceptible to cefepime, 29 with viridans streptococcal bacteremia not susceptible to cefepime).

**Table 5:** Multivariate analysis for risk factors for non-susceptibility to cefepime

**Factor**	**Odds ratio**	**95% confidence interval**	***p*-value**
Age	1.001	0.967 – 1.037	0.936
Complete remission status	0.674	0.285 – 1.595	0.369
Antibacterial prophylaxis			
None			0.074
Trimethoprim/sulfamethoxazole	2.010	0.152 – 26.588	0.596
Ciprofloxacin	0.396	0.041 – 3.872	0.426
Duration of neutropenia before the diagnosis of VSB	1.063	0.988 – 1.144	0.101

*VSB* viridans streptococcal bacteremia.

Medical records on the complete course of chemotherapy and antibacterial therapy for febrile neutropenia with antibiotics which have anti-pseudomonal effect since the diagnosis of hematologic malignancies were reviewed in 166 cases (124 adults, 42 children). Of the remaining cases, medical records of 16 patients who had been referred from other hospitals were not completely reviewed, and 17 patients who had been newly diagnosed with hematologic malignancies were excluded because they had no previous history of antibacterial therapy for febrile neutropenia. The interval from the diagnosis of hematologic malignancy to the diagnosis of VSB was a median of three months (IQR: 2–7). Among the 166 patients, 137 patients with VSB susceptible to cefepime and 29 patients with VSB not susceptible to cefepime received a median of one course (IQR: 1–2) and a median of two courses (IQR: 1–2) of antibacterial therapy for febrile neutropenia, respectively. The number of preceding antibacterial therapies for febrile neutropenia was not significantly different between patients with VSB susceptible and not susceptible to cefepime (Table 4).

## Discussion

We investigated the clinical and laboratory characteristics of VSB in febrile neutropenic patients with hematologic malignancies and the antibiotic susceptibilities of the viridans streptococci. The data were compared between adults and children and also in patients with VSB susceptible and not susceptible to cefepime.

VSB occurred most commonly in AML patients (72.7%), 12 days (IQR: 10–14) after the beginning of consolidation chemotherapy (57.9%), and six days (IQR: 4–8) after the onset of neutropenia. This pattern of VSB occurrence was consistent with previous reports [18]. While oral mucositis, a risk factor for VSB, occurred in roughly 60% of patients with VSB in previous reports [9,19], it occurred at a lower rate of 20.3% in this study. On the other hand, gastrointestinal symptoms were common in all patient groups, and cough was common in children. Considering that viridans streptococci are normal flora of the gastrointestinal and upper respiratory tracts as well as oral mucosa [20,21], and that mucosal damage can occur at these sites following chemotherapy or HCT, this was a predictable result. Since young children often cannot adequately complain of their oral and abdominal pain, and their parents or medical personnel might easily recognize objective symptoms, such as diarrhea and cough, the reported incidence of oral mucositis and abdominal pain might be lower in children than in adults. Other clinical and laboratory characteristics were not significantly different between adults and children, and the aforementioned symptoms occurred in less than one-third of patients. Therefore, we concluded that there were no distinctive characteristics to distinguish between VSB in adults and children.

The 12.9% occurrence rate of severe complications attributable to VSB was lower than that of previous reports, which showed an occurrence rate of 18-39%, and the 2.0% mortality attributable to VSB in this study was also lower than that of previous reports, which showed mortality up to 20% [8-10]. Although Martino *et al.*[11] reported a higher occurrence rate of severe complications and death due to VSB in children than in adults, the occurrence rate of severe complications and death attributable to VSB and overall mortality were not significantly different between adults and children in the present study. Previous researchers did not find significant factors related to a worse prognosis in children, and there have been few studies comparing the clinical characteristics and prognoses between adults and children. Comparisons between adults and pediatric patients with severe complications attributable to VSB showed that children more commonly complained of cough and had a longer duration between the beginning of the preceding therapy and the diagnosis of VSB, similar to the comparison between all adults and children with VSB. When comparing patients with severe complications attributable to VSB and those without severe complications in this study, there was no significant difference except for the peak CRP level within a week after the diagnosis of VSB. This had a low positive predictive value of 23% for the occurrence of severe complications. Thus, we were also unable to identify a definite factor that could help anticipate severe complications in VSB.

Antibiotic susceptibility rates to cefotaxime, cefepime, and erythromycin were lower in children than in adults. Although we performed both univariate and multivariate analyses to determine risk factors for decreased susceptibility to cefepime, no significant factors were found. Recurrent antibiotic use may be related to the increase in antibiotic resistance [22,23]; however, there was no difference between patients with VSB susceptible and not susceptible to cefepime in the number of antibacterial therapies for febrile neutropenia after previous conventional chemotherapy or HCT. The fact that the first-line antibiotic agent for patients with hematologic malignancies was cefepime or ceftazidime in most adults and piperacillin/tazobactam for almost all children in our hospital also supports the finding that previous antibiotic use is not related to decreased susceptibility to cefepime. We also analyzed the effect of prophylactic antibiotics on susceptibility to cefepime since ciprofloxacin, principally given to adults, has a limited effect on Gram positive bacteria [24,25], while TMP/SMX, principally given to children, has a satisfactory effect [25,26]. The effect of prophylactic antibiotics on decreased susceptibility to cefepime may be small since antibacterial prophylaxis has been reported to be unrelated to increased antibiotic resistance in a meta-analysis [27], and since patients in the present study received ciprofloxacin or TMP/SMX rather than β-lactam antibiotics and antibacterial prophylaxis with these antibiotics is not known to trigger antibiotic resistance in viridans streptococci [22,28,29]. Nevertheless, prophylactic antibiotic effects on decreased susceptibility to cefepime should not be ignored. Viridans streptococci can acquire β-lactam resistance through transfer of the mutated penicillin binding protein gene from *Streptococcus pneumoniae*[30,31], and it has been reported that the resistance of *S. pneumoniae* to β-lactam antibiotics after TMP/SMX prophylaxis in human immunodeficiency virus-infected patients can increase by a factor of 1.71 [32]. However, resistance to penicillin of *S. pneumoniae* was 0.3% in nonmeningeal isolates and 83.3% in meningeal isolates, and ceftriaxone resistance was 1.9% in nonmeningeal isolates and 0% in meningeal isolates from 2008 to 2009 in the Republic of Korea [33]. The exact effect of prophylactic antibiotics on the development of antibiotic resistance remains controversial [2], and the type of antibiotics, duration of prophylaxis, bacterial species, and host factors may influence the development of antibiotic resistance [22,34].

In this study, there were no definite differences in clinical and laboratory characteristics, mortality, or occurrence of severe complications between febrile neutropenic adults and children with VSB, despite a significant difference in antibiotic susceptibility to cefepime between the two groups. Antibiotic susceptibilities were not significantly related to the development of severe complications. Thus, our study results show that different antibiotic treatment strategies for adults and children with VSB are not necessary. The lower susceptibility rate of 66.1% to cefepime in children may indicate the need for initial glycopeptide therapy in febrile neutropenic children. However, bacteremia is diagnosed in 10-25% of febrile neutropenic children [1-3], and 20-30% of the bacteremia is caused by viridans streptococci [1,4,5]. In addition, since severe complications occurred in 6.6% of the patients with VSB according to our results, we estimate that the incidence of severe complications of VSB in febrile neutropenic children is 0.5%. Therefore, considering that antibiotic susceptibility is not significantly related to the prognosis of VSB in febrile neutropenia [5,9], universal initial glycopeptide therapy targeting only 0.5% of febrile neutropenic children with hematologic malignancies should not be considered. Instead, we should consider glycopeptide therapy if antibiotic susceptibility tests revealed that the isolated viridans streptococci were not susceptible to antibiotics being administered to the patient and susceptible to glycopeptides.

This study has several limitations including its retrospective nature. We tried to eliminate selection bias by including all consecutive hematologic malignancy patients with VSB who were treated in the same hospital environment. Also, there were some limitations in our tests for antibiotic susceptibility. The results of the E-test and disk diffusion method for antibiotic susceptibility in this study may be different from results of broth microdilution methods. Additionally the clinical laboratory of our hospital did not perform piperacillin/tazobactam susceptibility test for viridans streptococci; thus, we assumed that cefepime susceptibility was similar to piperacillin/tazobactam susceptibility. This assumption may not be applicable to clinical settings. Lastly, past histories of antibacterial therapy for febrile neutropenia were reviewed to evaluate its effect on the differences in antibiotic susceptibility; however, information from 35 patients was missing. Although we assumed that previous antibacterial therapies should not influence β-lactam susceptibilities, the relationship should be further investigated.

## Conclusions

In this study, no definite differences in clinical and laboratory characteristics or prognosis were found between febrile neutropenic adults and children with VSB. While susceptibility to cefepime was lower in children, there were no differences in clinical characteristics or prognosis between patients with VSB susceptible and not susceptible to cefepime. Therefore, this study showed that different antibiotic treatment strategies for adults and children with VSB are not necessary, and also confirmed that current IDSA and Korean guidelines for febrile neutropenic patients may be applied to both febrile neutropenic children and adults with VSB. Further studies on the cause and clinical significance of the difference in antibiotic susceptibility rates between adults and children are needed.

## Competing interests

There is no competing interest for any authors.

## Authors’ contributions

SBH, DGL, BC, and JHK designed this study. SBH, EYB and JWL collected data, and NGC and DCJ analysed the data. SBH, JWL and DGL wrote the manuscript, and BC, JHK and HKK critically reviewed the manuscript. All authors read and approved the final draft.

## Pre-publication history

The pre-publication history for this paper can be accessed here:

http://www.biomedcentral.com/1471-2334/13/273/prepub
